# Aging brain: exploring the interplay between bone marrow aging, immunosenescence, and neuroinflammation

**DOI:** 10.3389/fimmu.2024.1393324

**Published:** 2024-04-04

**Authors:** Ludmila Müller, Svetlana Di Benedetto

**Affiliations:** Max Planck Institute for Human Development, Center for Lifespan Psychology, Berlin, Germany

**Keywords:** aging, brain, bone marrow, neuroinflammation, immunosenescence, inflammaging, neurological disorders

## Abstract

Aging is a complex process characterized by a myriad of physiological changes, including alterations in the immune system termed immunosenescence. It exerts profound effects on both the bone marrow and the central nervous system, with significant implications for immunosenescence in neurological contexts. Our mini-review explores the complex relationship between bone marrow aging and its impact on immunosenescence, specifically within the context of neurological diseases. The bone marrow serves as a crucial hub for hematopoiesis and immune cell production, yet with age, it undergoes significant alterations, including alterations in hematopoietic stem cell function, niche composition, and inflammatory signaling. These age-related shifts in the bone marrow microenvironment contribute to dysregulation of immune cell homeostasis and function, impacting neuroinflammatory processes and neuronal health. In our review, we aim to explore the complex cellular and molecular mechanisms that link bone marrow aging to immunosenescence, inflammaging, and neuroinflammation, with a specific focus on their relevance to the pathophysiology of age-related neurological disorders. By exploring this interplay, we strive to provide a comprehensive understanding of how bone marrow aging impacts immune function and contributes to the progression of neurological diseases in aging individuals. Ultimately, this knowledge can hold substantial promise for the development of innovative therapeutic interventions aimed at preserving immune function and mitigating the progression of neurological disorders in the elderly population.

## 1 Introduction

Aging is a multifaceted process characterized by progressive declines in physiological function and increased susceptibility to disease. One of the hallmarks of aging is immunosenescence - the gradual deterioration of the immune system, which significantly impacts an individual’s ability to respond to pathogens and maintain tissue homeostasis (1–5). In parallel, aging also affects the central nervous system (CNS), leading to alterations in cognitive function, neuronal health, and susceptibility to neurological diseases (6–9). Understanding the interplay between aging, immunosenescence, and neurological health is of paramount importance in addressing the growing burden of age-related neurological disorders.

The bone marrow serves as a central hub for hematopoiesis, generating a diverse array of immune cells essential for host defense and immune surveillance (10–12). However, with advancing age, the bone marrow undergoes profound changes, including alterations in hematopoietic stem cell function, shifts in immune cell composition, and dysregulation of inflammatory signaling pathways. These age-related changes in the bone marrow microenvironment have significant implications for immune cell production, differentiation, and function, ultimately contributing to the development of immunosenescence (11, 13, 14).

Concurrently, aging also impacts the CNS, leading to neuroinflammatory processes, synaptic dysfunction, and neuronal damage (15–18). Emerging evidence suggests that immunosenescence plays a pivotal role in driving neuroinflammation and exacerbating neuronal dysfunction in age-related neurological diseases such as Alzheimer’s disease, Parkinson’s disease, and multiple sclerosis (15, 17, 19, 20). The collective alterations witnessed in the aging immune system, coupled with the impact of pro-inflammatory immune factors in exacerbating brain dysfunction, underscore the crucial role of the aging hematopoietic system as a significant driver of brain aging. Evidence suggests that inflammatory insults can induce a persistent increase in markers of hematopoietic stem cell (HSC) aging, even in young mice, underscoring the profound impact of immune and inflammatory processes on the aging brain (21–23). Furthermore, heterochronic HSC transplantations, involving the reconstitution of young mice’s bone marrow with aged HSCs, resulted in decreased hippocampal neurogenesis, impaired cognitive functions, and reduced synaptic density in adult recipient mice (21, 24).

Research on heterochronic parabiosis has demonstrated its ability to mitigate various cellular hallmarks of aging in the brain. This includes enhancements such as increased synaptic plasticity and density, amplified hippocampal neurogenesis, elevated vascular density and cerebral blood flow, as well as reduction in cellular senescence markers within the forebrain (25–27). Similarly, replenishing the cellular composition of aged blood with young immune cells via heterochronic bone marrow transplantation led to elevated synaptic density, reduced microglial activation, and improved hippocampus-dependent cognitive function in aged mice (21, 28, 29). Preclinical and clinical investigations are currently underway to evaluate the efficacy of approaches involving young blood and plasma exchange in addressing aging-associated neurodegenerative diseases, including Alzheimer’s disease (30, 31).

Therefore, it is pivotal to elucidate the complex underlying mechanisms by which aging of the bone marrow exerts its influence on the progression of immunosenescence, and subsequently, to delineate how immunosenescence contributes to the pathogenesis and exacerbation of neurological diseases. This mini-review aims to explore the impact of aging on neurological immunosenescence, with a specific focus on the role of bone marrow aging in shaping immune function and neuroinflammation. By elucidating the interplay between bone marrow aging, immunosenescence, and neurological heath we can gain insights into potential therapeutic strategies aimed at preserving immune function and ameliorating neurological disease progression in the aging population.

## 2 Bone marrow as a hub for immune cell generation: impact of aging

The bone marrow stands as a pivotal organ in the human body, serving as a primary site for hematopoiesis and the production of immune cells essential for maintaining host defense and immune surveillance (4, 12, 32). Within the bone marrow microenvironment, hematopoietic stem cells reside in specialized niches, where they undergo self-renewal and differentiation processes to generate a diverse array of blood and immune cell lineages. Through a tightly regulated process, HSCs give rise to multipotent progenitor cells that subsequently differentiate into lineage-committed precursors, ultimately producing mature immune cells, including lymphocytes (T- and B cells), myeloid cells (monocytes, macrophages, dendritic cells), and erythrocytes. This continuous generation of immune cells within the bone marrow ensures the replenishment of circulating immune cell populations, enabling the body to mount effective immune responses against pathogens, clear damaged cells, and maintain tissue homeostasis (10, 12, 33). Thus, the bone marrow serves as a critical reservoir for immune cell production, playing an indispensable role in orchestrating immune responses and preserving overall health and well-being.

**Figure 1:**
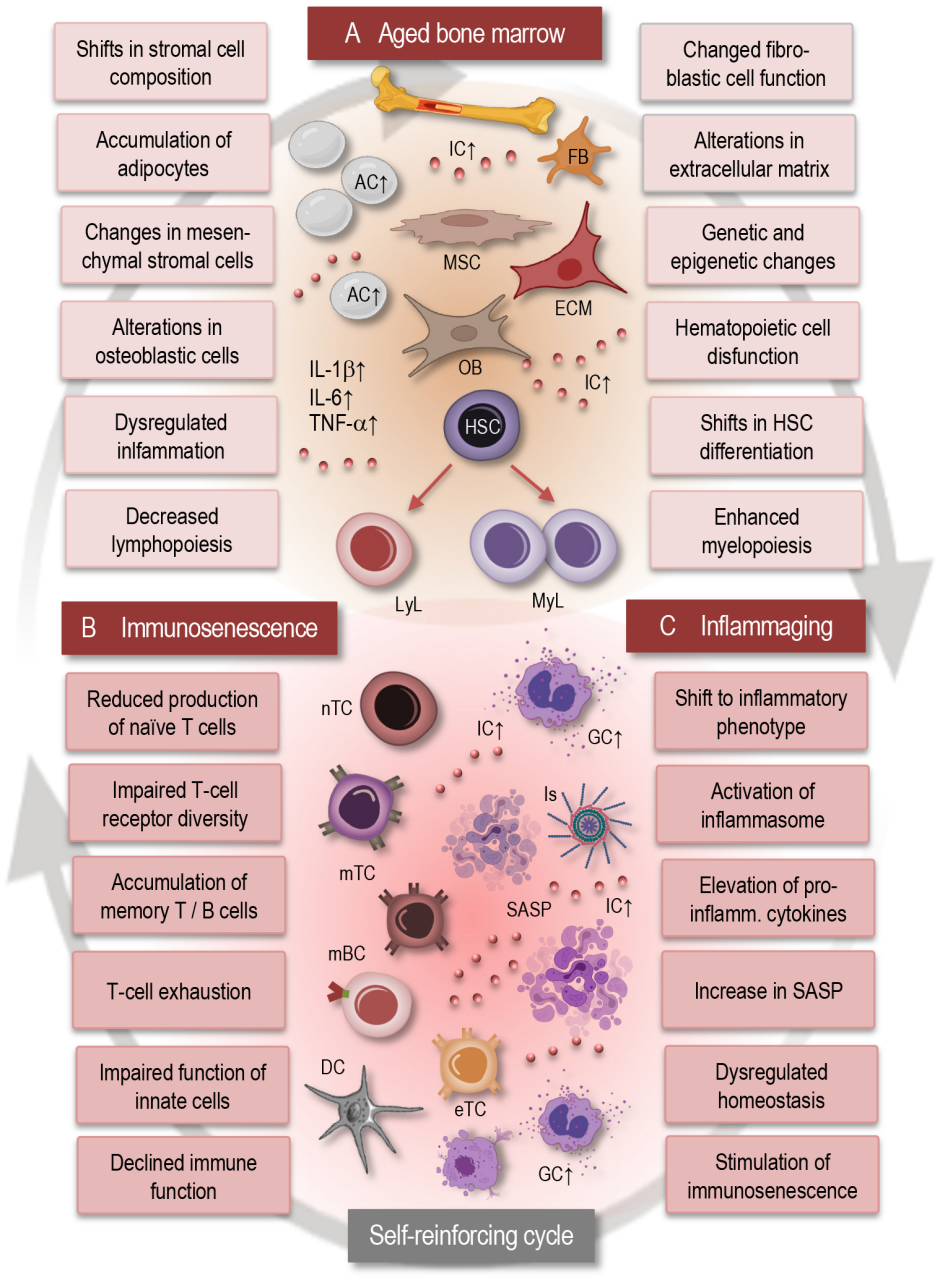
Interplay between bone marrow aging, immunosenescence, and inflammaging. This simplified scheme illustrates the dynamic interactions between bone marrow aging **(A)**, immunosenescence **(B)**, and inflammaging **(C)**. Aging of the bone marrow microenvironment leads to alterations in HSC function and differentiation, contributing to immunosenescence characterized by decreased immune cell diversity and function. Concurrently, inflammaging promotes a pro-inflammatory environment within the bone marrow, further exacerbating immunosenescence. This self-reinforcing cycle between bone marrow aging, immunosenescence, and inflammaging contributes to age-related immune dysregulation and increased susceptibility to age-related disorders. AC, adipocytes; IC, inflammatory cytokines; MSC, mesenchymal stromal cell; FB, fibroblast; OB, osteoblast; ECM, extracellular matrix; GC, granulocyte; LyL, lymphatic lineage; MyL, myeloid lineage; HSC, hematopoietic stem cell; nTC, naïve T cell; mTC, memory T cell; mBC, memory B cell; Is, inflammasome; eTC, exhausted T cell; SASP, senescence associated phenotype; IL, interleukin; TNF, tumor necrosis factor.

With advancing age, the bone marrow microenvironment undergoes remarkable alterations that profoundly influence hematopoiesis and immune cell function (**Figure 1A**). These changes encompass a spectrum of cellular and molecular modifications within the bone marrow niche, including shifts in stromal cell composition, increased adiposity, and dysregulated signaling pathways (4, 34). Age-related alterations in stromal cell populations, such as mesenchymal stromal cells (MSCs) and osteoblasts, modulation of fibroblastic reticular cell populations, changes in the extracellular matrix composition and organization disrupt the supportive microenvironment necessary for HSC maintenance and differentiation. Moreover, an accumulation of adipocytes within the bone marrow, known as adipogenesis, displaces hematopoietic and stromal cells, leading to diminished hematopoietic activity and impaired immune cell production (11, 13, 33–35).

Dysregulated signaling pathways, including increased production of pro-inflammatory cytokines and altered expression of niche factors, further contribute to the disruption of hematopoiesis and immune cell homeostasis in the aging bone marrow microenvironment. Collectively, these age-related alterations in the bone marrow microenvironment contribute to immune dysfunction and immunosenescence, highlighting the complex interplay between the aging bone marrow and systemic immune aging (12, 36, 37).

Hematopoietic stem cell dysfunction represents a critical component of bone marrow aging, significantly impacting immune cell production and overall hematopoietic activity (4, 12, 34, 38). As individuals age, HSCs undergo alterations in their self-renewal capacity and differentiation potential, leading to a decline in the generation of diverse immune cell lineages (**Figure 1A**). This age-related decline in HSC function manifests as impaired lymphopoiesis, resulting in reduced output of naïve T- and B cells, alongside enhanced myelopoiesis, leading to increased production of myeloid cells such as monocytes and granulocytes. The dysregulation of HSC function not only disrupts the balance between different immune cell populations but also compromises the ability of the immune system to support effective responses to pathogens and maintain immune surveillance. Moreover, accumulated genetic and epigenetic changes in HSCs with age further contribute to their dysfunction, increasing the susceptibility to hematologic malignancies and immune-related disorders (36, 39–41).

Bone marrow aging is closely associated with alterations in the hematopoietic microenvironment, which can contribute to the secretion of inflammatory factors such as interleukin-6 (IL-6), tumor necrosis factor-alpha (TNF-α), and interleukin-1β (IL-1β). Changes in inflammatory signaling pathways within the bone marrow microenvironment play a pivotal role in driving immune dysregulation during aging.

Several causal links may exist between bone marrow aging and the increased production of these inflammatory cytokines (11, 36, 38):

*Dysregulation of hematopoiesis*: As previously noted, the bone marrow microenvironment experiences notable alterations with aging, including changes in the composition of stromal cells and extracellular matrix components. These changes can disrupt the balance of HSC niches, leading to dysregulated hematopoiesis and increased production of myeloid lineage cells. Myeloid cells, such as monocytes and macrophages, are major producers of inflammatory cytokines like IL-6, IL-1β, and TNF-α.

*Senescent cell accumulation*: Aging is associated with the accumulation of senescent cells in various tissues, including the bone marrow. Senescent hematopoietic and stromal cells within the bone marrow can contribute to the local production of IL-6, IL-1β, and TNF-α.

*Increased oxidative stress*: Aging is also characterized by increased oxidative stress within the bone marrow microenvironment. Elevated levels of reactive oxygen species (ROS) can activate signaling pathways involved in the production of inflammatory cytokines. For example, ROS-mediated activation of nuclear factor kappa B (NF-κB) signaling can lead to enhanced expression of pro-inflammatory cytokines such as IL-6, IL-1β, and TNF-α.

*Chronic low-grade inflammation*: With advancing age, there is a shift towards a pro-inflammatory state characterized by increased production of inflammatory cytokines, which affects multiple tissues including the bone marrow. Inflammaging can result from the cumulative effects of cellular senescence, oxidative stress, and dysregulated immune responses. Inflammatory cytokines produced within the bone marrow contribute to the systemic inflammatory milieu observed in aging and can further perpetuate inflammation in distant tissues.

Overall, the causal links between bone marrow aging and the secretion of inflammatory factors involve dysregulation of hematopoiesis, accumulation of senescent cells, increased oxidative stress, and the presence of chronic low-grade inflammation. These mechanisms collectively contribute to the elevated production of IL-6, IL-1β, and TNF-α within the aging bone marrow microenvironment, with implications for systemic inflammation and age-related diseases.

Heightened inflammatory signaling not only alters the behavior of HSCs but also influences the differentiation and function of various immune cell subsets. Specifically, inflammatory cytokines promote the expansion of pro-inflammatory immune cell populations, such as myeloid-derived suppressor cells (MDSCs), while inhibiting the differentiation and function of anti-inflammatory immune cells, including regulatory T cells (Tregs) and M2 macrophages (42, 43).

In fact, inflammaging fosters a proinflammatory microenvironment in the bone marrow, triggering inflammatory signaling cascades that perpetuate further inflammatory and senescent alterations in the immune system, thereby establishing a self-reinforcing cycle (**Figure 1**). Consequently, dysregulated inflammatory signaling exacerbates age-related immune dysfunction including neuroinflammation and contributes to the pathogenesis of various age-associated diseases and neurological disorders (11, 44–47). Understanding this complex interplay between inflammatory signaling and immune dysregulation within the bone marrow microenvironment is crucial for unraveling the mechanisms of the age-related immune pathologies.

In conclusion, the bone marrow serves as a critical site for hematopoiesis and immune cell production, while also playing a key role in regulating systemic inflammation and immune surveillance. With advancing age, the bone marrow undergoes significant alterations in its composition and function, including changes in HSC function, alterations in immune cell production, and dysregulation of inflammatory signaling pathways. These age-related changes in the bone marrow microenvironment and in the HSC function have profound implications for immune cell homeostasis and function, ultimately contributing to the development of immunosenescence.

## 3 Aging immune system: the impact of bone marrow aging on immunosenescence

Immunosenescence, the gradual deterioration of the immune system with aging, is a phenomenon that has gained increasing attention in the context of neurological diseases. As individuals age, their immune system undergoes significant changes, including alterations in the composition and function of immune cells, dysregulation of inflammatory signaling, and impaired immune responses (4, 32, 48, 49). These age-related changes in immune function have profound implications for neurological health, as the immune system plays a crucial role in maintaining brain homeostasis, modulating neuroinflammatory processes, and protecting against neurodegenerative diseases.

The hallmarks of immunosenescence comprise alterations in both the innate and adaptive immune compartments and include the age-related thymic involution - the progressive decline in thymic function with age, which leads to reduced production of naïve T cells and impaired T-cell receptor diversity. This age-related decline in thymic output compromises the adaptive immune response to new pathogens and reduces the pool of antigen-inexperienced T cells (50–52).

In general, bone marrow aging exerts a significant impact on the aging of the adaptive immune system, leading to alterations in T- and B- cell development, function, and homeostasis (34). Immunosenescence (**Figure 1B**) is characterized by an accumulation of memory T cells, particularly effector memory T cells (TEM) and terminally differentiated memory T cells (TEMRA). This shift in T-cell populations reflects a history of antigen exposure and may contribute to chronic inflammation and immune dysfunction (4, 53). Chronic antigenic stimulation and persistent infections may lead to T-cell exhaustion, characterized by decreased proliferation, cytokine production, and cytotoxic activity, contributing to impaired immune responses in older individuals. Immunosenescence influences the differentiation of T cells, thus leading to alterations in the balance between regulatory T cells, effector T cells, and memory T cells (3, 4, 54, 55). Dysregulated T-cell differentiation may contribute to impaired immune surveillance, chronic inflammation, and to autoimmune and neurogenerative disorders. Immunosenescence and bone marrow aging are also associated with a decline in B-cell function, including reduced antibody production, impaired class switching, and decreased affinity maturation (56, 57).

Additionally, bone marrow aging significantly impacts innate immune cells, leading to alterations in their development, function, and behavior. This process includes impairments in the differentiation and function of macrophages and dendritic cells leading to diminished phagocytosis, antigen presentation, and altered cytokine production. Immunosenescence is accompanied by alterations in innate immune cell function, dysregulated inflammasome activation, and reduced tissue repair and regeneration (10, 58, 59). Age-related changes in the bone marrow microenvironment can skew the differentiation of precursor cells towards a pro-inflammatory phenotype, contributing to chronic inflammation, tissue damage, and impaired immune responses (11, 33, 36). The development and function of natural killer (NK) cells are also compromised, leading to alterations in their cytotoxic activity and cytokine production as well as in their ability to eliminate infected or transformed cells and to contribute to immune surveillance (4, 13, 34, 60). Aged bone marrow microenvironment can lead to alterations in granulocyte production, differentiation, and migration, affecting their ability to respond to pathogens and contribute to inflammatory processes (4, 13).

Generally, aging of the immune system is associated with a chronic low-grade inflammatory state, commonly referred to as inflammaging (**Figure 1C**), which is characterized by elevated levels of pro-inflammatory cytokines such as IL-6, IL-1β, and TNF-α (45, 61). This inflammatory imbalance may disrupt immune homeostasis and contribute to age-related pathologies, including neurodegeneration, cardiovascular and autoimmune disorders (8, 62, 63). Cellular senescence, characterized by irreversible growth arrest and altered secretory phenotype, contributes to immunosenescence by promoting chronic inflammation and tissue damage. Senescent immune cells, including senescent T cells and senescent macrophages, accumulate with age, promote inflammaging, and contribute to age-related pathologies (19, 64–66).

Importantly, senescence-associated secretory phenotype (SASP) refers to the unique secretory profile exhibited by senescent cells and is characterized by the release of various pro-inflammatory cytokines, chemokines, growth factors, and extracellular matrix remodeling enzymes (66). This complex mixture of factors contributes to the chronic low-grade inflammation observed in aging tissues and plays a significant role in driving age-related pathologies. The SASP can induce tissue remodeling, facilitate immune surveillance, and stimulate senescence in neighboring cells through paracrine signaling. Moreover, chronic exposure to SASP factors can also fuel chronic inflammation, disrupt tissue homeostasis (**Figure 1C**), and contribute to the development of age-related diseases, including cancer and neurodegenerative disorders (37, 66).

In recent years, there has been growing recognition of the impact of immunosenescence on the pathogenesis and progression of neurological disorders such as Alzheimer’s disease, Parkinson’s disease, and multiple sclerosis (14, 15, 66, 67). Understanding the interplay between immunosenescence and neurological diseases is essential for elucidating disease mechanisms, developing interventions to preserve cognitive function and promote healthy aging. In the following sections we will set the stage for exploring the complex relationship between immunosenescence and neurological diseases, highlighting the importance of immune health in maintaining brain function throughout the aging process.

## 4 Interplay between the immune and nervous systems in health and diseases

**Figure 2:**
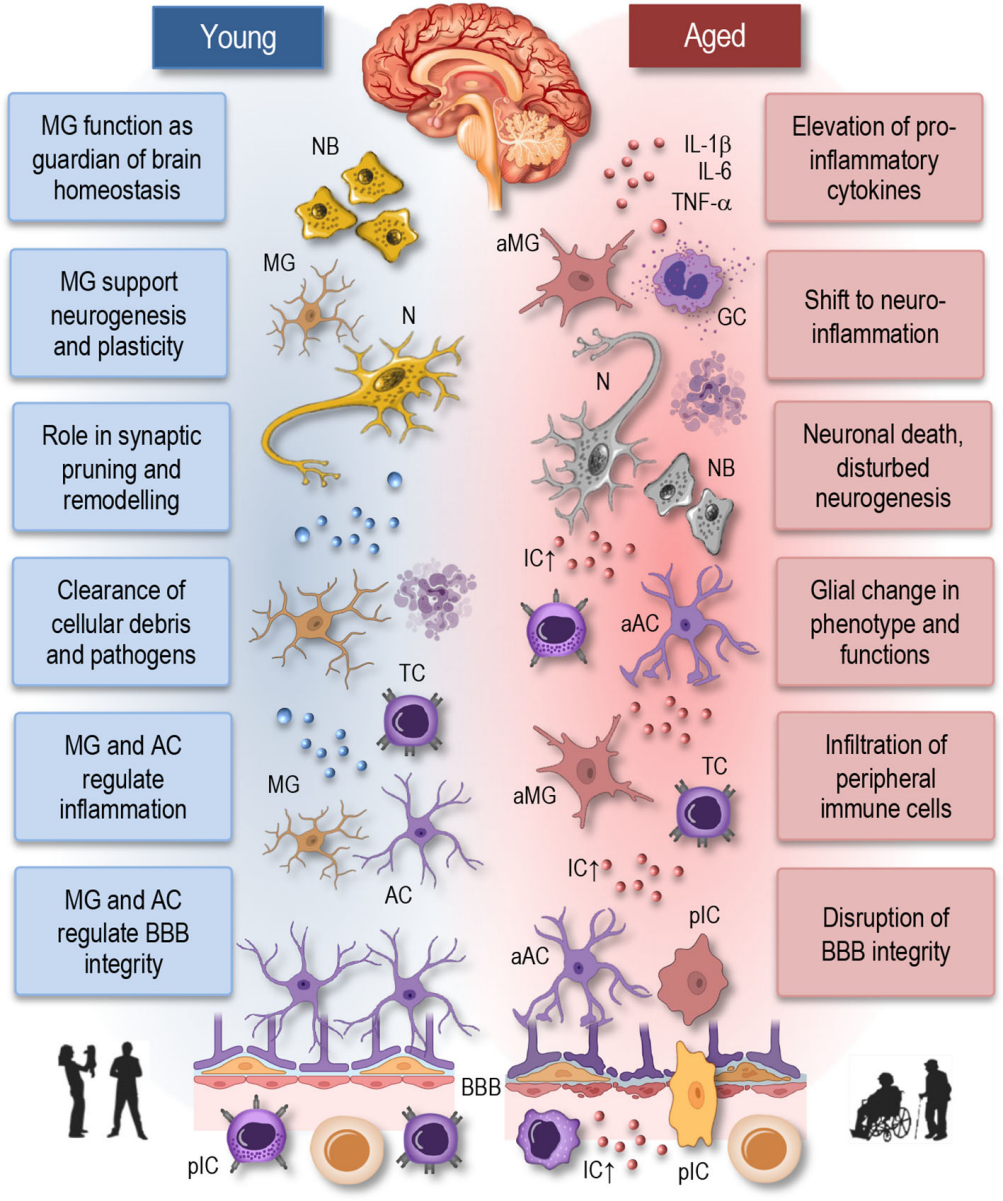
Neuroimmune interactions in young and aged brain. This simplified illustration depicts the neuroimmune interactions in the young (homeostatic) and aged brain. In the young brain (left panel), there is a delicate balance between immune cells, including microglia and astrocytes, and neurons, maintaining homeostasis and supporting neuronal function. Microglia actively survey the brain parenchyma and respond to neuronal activity, pathogens, or injury by adopting various phenotypic states, ranging from surveillant to activated. Astrocytes play critical roles in synaptic function, neuroprotection, and maintaining the blood-brain barrier integrity. In contrast, the aged brain (right panel) exhibits dysregulated neuroimmune interactions characterized by increased microglial activation, astrocyte reactivity, and pro-inflammatory cytokine release. Disruption of the blood-brain barrier facilitates the infiltration of immune cells into the brain parenchyma, contributing to neuroinflammation and neuronal death. These alterations contribute to neuronal dysfunction and age-related neurological disorders. NB, neuroblasts; MG, microglia; aMG, activated microglia; N, neuron; TC, T cell; AC, astrocyte; aAC, activated astrocyte; BBB, blood-brane-barrier; IL, interleukin; TNF, tumor necrosis factor; IC, inflammatory cytokines; GC, Granulocyte; pIC, peripheral immune cells.

The immune system serves as a sentinel and guardian of brain health, orchestrating a complex array of responses to maintain the delicate balance within the CNS (**Figure 2**, left). Central to this role are microglia, the resident immune cells of the brain, which constantly surveil the CNS environment for signs of injury, infection, or aberrant cellular activity (67, 68). Through a process of vigilant monitoring, microglia promptly respond to any disruptions in brain homeostasis, including the clearance of cellular debris, the phagocytosis of pathogens, and the regulation of neuroinflammatory processes. Importantly, the immune system’s involvement in synaptic pruning and remodeling during development is essential for sculpting neural circuits and optimizing synaptic connectivity, thereby supporting cognitive function and plasticity (17, 69). Moreover, immune cells collaborate with the neurovascular unit to maintain the integrity of the blood-brain barrier (BBB), ensuring selective permeability and shielding the CNS from potentially harmful substances circulating in the bloodstream. Age-related dysfunction in immune responses within the brain can lead to chronic neuroinflammation, synaptic dysfunction, and neuronal damage, which are hallmark features of various neurological disorders.

Astrocytes, the most abundant glial cells in the brain, play a pivotal role in regulating neuroinflammation (**Figure 2**, left). Once considered merely supportive cells, astrocytes are now recognized as dynamic regulators of immune responses within the CNS (70). Upon encountering inflammatory stimuli, such as infection, injury, or neurodegeneration, astrocytes become activated and undergo significant phenotypic changes. Activated astrocytes release a variety of pro-inflammatory and anti-inflammatory mediators, including cytokines, chemokines, growth factors, and reactive oxygen species, which influence the activity of neighboring neurons, microglia, and endothelial cells (17, 70–73). Additionally, astrocytes modulate the blood-brain barrier integrity, regulate synaptic function, and participate in the clearance of extracellular debris and pathogens. However, dysregulated astrocyte activation can exacerbate neuroinflammation and contribute to neuronal damage and disease progression.

Remarkably, apart from immune cells, a variety of other cell types such as mesenchymal stromal/stem cells, fibroblasts, endothelial cells, osteoblasts, neurons, and Schwann cells demonstrate notable immune functions. These functions include the secretion of cytokines, chemokines, and growth factors, as well as the facilitation of inflammation, antigen presentation, immunosuppression, and antimicrobial effects, particularly in response to conditions like infection and inflammation (70, 74). Aging processes in these cells may lead to functional impairments, resulting in disturbances in their activity and function, thus contributing to age-related diseases.

The complex interplay between the immune and nervous systems manifests additionally in the concept of neuroimmune cell units, where immune cells closely interact with neurons and glial cells, shaping the dynamics of neuroinflammation and influencing disease progression in various neurological disorders (74, 75). In this regard, neuroimmune cell units are emerging as pivotal coordinators of diverse physiological processes, encompassing hematopoiesis, inflammatory responses, and tissue regeneration (76). These specialized structures facilitate direct communication and coordination between the two systems, with immune cells such as microglia, B- and T cells, dendritic cells, and macrophages interacting closely with neurons and other glial cells. These interactions, occurring through physical contacts or synapse-like connections, involve the exchange of signaling molecules, including cytokines, chemokines, neurotransmitters, and other immune modulators (7, 74). Neuroimmune cell units are believed to exert a significant influence on regulating neuroinflammatory responses, modulating neuroimmune signaling, and impacting disease progression in various neurological disorders (74).

Thus, the immune system plays a multifaceted and indispensable role in sustaining brain health and function throughout life. Therefore, it is reasonable to anticipate that age-related alterations in immune cell function and neuroinflammatory processes will significantly contribute to the pathogenesis and progression of neurological diseases (67, 70). Indeed, as individuals age, the immune system undergoes profound changes, that impact both peripheral immune cells and resident immune cells within the CNS, including microglia (77, 78). Aging microglia undergo phenotypic and functional changes, characterized by a shift towards a pro-inflammatory state, commonly referred to as “primed” or “senescent” microglia (**Figure 2**, right). Primed microglia exhibit exaggerated responses to stimuli and produce higher levels of pro-inflammatory cytokines, thus contributing to chronic neuroinflammation (8, 18, 73). This sustained neuroinflammatory state is detrimental to neuronal health and has been implicated in the pathogenesis of various neurological disorders, including Alzheimer’s disease, Parkinson’s disease, and multiple sclerosis (14, 17, 20, 58, 67, 69, 70).

Furthermore, age-related changes in the BBB-permeability and neurovascular dysfunction exacerbate neuroinflammation (**Figure 2**, right), allowing the infiltration of peripheral immune cells into the CNS (8). Peripheral immune cells, including monocytes and T cells, contribute to neuroinflammatory processes and further exacerbate neuronal damage and cognitive decline (20, 58, 69). Age-related changes in immune cell function, including impaired phagocytosis and antigen presentation, may compromise immune surveillance within the CNS, leading to reduced clearance of protein aggregates and cellular debris. This impaired immune surveillance contributes to the accumulation of neurotoxic substances and the development of neurodegenerative diseases (8, 15, 19, 79).

The exploration of the interaction between astrocytes and immune cells derived from the bone marrow unveils a complex interplay vital for understanding neuroinflammation linked to aging. The relationship between astrocytes and aging immune cells is a dynamic and complicate process that plays a crucial role in the maintenance of CNS homeostasis and the pathogenesis of age-related neuroinflammatory disorders (80, 81). Astrocytes, the most abundant glial cells in the CNS, undergo significant changes with aging, leading to alterations in their morphology, gene expression, and functional properties (81, 82). Concurrently, aging is associated with dysregulation of the immune system, characterized by alterations in immune cell populations, increased production of pro-inflammatory cytokines, and impaired immune responses (72).

In the context of aging, astrocytes actively engage with immune cells infiltrating the CNS, including microglia, monocytes, and T cells. Astrocytes can respond to signals from aging immune cells by altering their phenotype and secretory profile. For instance, aged microglia and peripheral immune cells secrete pro-inflammatory cytokines such as IL-6, TNF-α, and IL-1β. In response to these inflammatory cues, astrocytes become activated and produce cytokines, chemokines, and reactive oxygen species, which contribute to the establishment of a neuroinflammatory environment in the aging brain (80, 81, 83, 84).

Conversely, astrocytes also exert regulatory effects on aging immune cells, influencing their activation state, migration, and function. Astrocyte-derived factors such as transforming growth factor-beta (TGF-β), IL-10, and prostaglandin E2 (PGE2) can exert immunosuppressive effects on immune cells, dampening excessive inflammation and promoting tissue repair (85–87). However, dysregulated astrocyte activation in the aging brain can lead to aberrant immune responses, exacerbating neuroinflammation and neuronal damage (88, 89).

Furthermore, the bidirectional communication between astrocytes and aging immune cells can have profound implications for CNS function and dysfunction in aging. Chronic neuroinflammation driven by dysregulated astrocyte-immune cell interactions is implicated in the pathogenesis of age-related neurodegenerative diseases such as Alzheimer’s disease, Parkinson’s disease, and multiple sclerosis (80, 81, 85, 90). Understanding the interplay between astrocytes and aging immune cells is essential for elucidating the mechanisms underlying age-related neuroinflammation and may offer novel therapeutic targets for mitigating neurodegenerative disorders in the aging population.

Thus, age-related dysregulation of immune cell production and function in the periphery may result in an altered immune response within the CNS, leading to chronic neuroinflammation and neuronal damage (8, 17). Conversely, neuroinflammation in the CNS can also impact immune cell function, potentially exacerbating age-related changes in hematopoiesis and immune cell production. Moreover, emerging evidence suggests bidirectional communication between the bone marrow and the CNS, mediated by factors such as cytokines, chemokines, and extracellular vesicles (11, 74). These communication pathways may play a crucial role in coordinating immune responses between the bone marrow and the CNS, influencing the progression of neuroinflammation and neurological diseases.

Overall, the interplay between age-related changes in immune cell function and neuroinflammatory processes plays a crucial role in the pathophysiology of neurological diseases, thus highlighting its significance as a therapeutic target for promoting cognitive resilience and combating neurological diseases. Targeting these processes represents a promising therapeutic approach for mitigating neuroinflammation and preserving neuronal function in the aging population. However, further research is needed to reveal the underlying mechanisms and identify novel therapeutic targets for effective intervention.

## 5 Future directions

The elucidation of the complex interplay between bone marrow aging, immunosenescence, and neuroinflammation in neurological diseases opens avenues for future research aimed at developing innovative therapeutic strategies and advancing our understanding of disease pathophysiology. Future studies should aim to unravel the underlying mechanisms linking bone marrow aging to neuroinflammation and neuronal dysfunction in neurological diseases. Understanding the cellular and molecular pathways involved in immune dysregulation and neuroinflammatory processes will provide valuable insights into disease pathogenesis and identify novel therapeutic targets. Several key areas warrant further investigation:

*Biomarker discovery*: Identification of biomarkers of immune dysregulation and neuroinflammation holds promise for early disease detection, prognostication, and monitoring treatment response in neurological diseases. Biomarker discovery efforts should focus on elucidating circulating factors, genetic signatures, and imaging markers indicative of immune dysfunction and neuroinflammation.

*Targeted therapies*: Development of targeted therapeutic strategies aimed at modulating immune function and neuroinflammatory processes represents a critical direction for future research. Efforts should focus on optimizing existing immunomodulatory therapies, exploring novel therapeutic targets, and developing innovative drug delivery systems to enhance treatment efficacy and minimize off-target effects.

*Precision medicine approaches*: Integration of precision medicine approaches into clinical practice will enable personalized therapeutic interventions tailored to individual patient profiles, including genetic, epigenetic, and immunological factors. Precision medicine strategies should incorporate multi-omic data integration, computational modeling, and artificial intelligence algorithms to optimize treatment selection and improve patient outcomes.

*Translational research*: Translation of preclinical findings into clinical applications is essential for advancing therapeutic development and improving patient care. Collaborative efforts between basic scientists, clinicians, and industry partners are needed to facilitate the translation of promising therapeutic candidates from bench to bedside, accelerating the development of effective treatments for neuroimmune disorders and neurological diseases.

## 6 Conclusions

In conclusion, the interplay between bone marrow aging, immunosenescence, and neuroinflammation plays a pivotal role in the pathogenesis and progression of neurological diseases. Age-related changes in the bone marrow microenvironment may contribute to immune dysregulation and chronic neuroinflammation, leading to neuronal dysfunction and cognitive decline in aging populations. Despite the challenges posed by the complex nature of these interactions, ongoing research efforts hold promise for the development of innovative therapeutic strategies aimed at preserving neuronal health and function. By elucidating the underlying mechanisms of bone marrow aging, immune dysregulation, and neuroinflammation and translating these findings into clinical practice, we can advance our understanding of disease pathophysiology and improve outcomes for patients with neurodegenerative diseases.

## Author contributions

LM: Conceptualization, Supervision, Visualization, Writing – original draft, Writing – review & editing. SD: Conceptualization, Methodology, Writing – original draft, Writing – review & editing.
